# Knowledge, Awareness, and Perception of COVID-19 and Artificial Intelligence: A Cross-Sectional Study Among the Population in Saudi Arabia

**DOI:** 10.7759/cureus.40921

**Published:** 2023-06-25

**Authors:** Shaista Haleem, Nassreen H Albar, Yara S Al Fahhad, Hala O AlWasem

**Affiliations:** 1 Aesthetic and Restorative Dentistry, Riyadh Elm University, Riyadh, SAU; 2 Restorative Dentistry, Jazan University, Jazan, SAU; 3 Dentistry, King Saud University, Riyadh, SAU; 4 Dentistry, Riyadh Elm University, Riyadh, SAU

**Keywords:** artificial intelligence, covid-19, machine learning, saudi arabia, survey

## Abstract

Background: Artificial intelligence (AI) has made significant contributions to the development of medicines and vaccines. In addition, AI can analyze large amounts of COVID-19 test data, including the number of positive cases, to forecast the trajectory of the pandemic.

Aim: This study aimed to assess the knowledge, perception, and awareness of the general population in Saudi Arabia regarding AI and its application in combating COVID-19.

Methods: A cross-sectional research design was employed, and online surveys were distributed via email and social media platforms. Purposeful sampling was used to select participants who met the inclusion criteria. The reliability and validity of the survey instrument were also assessed.

Results: The majority of respondents (34.6%) fell within the age range of 30 to 39 years. The sample predominantly consisted of female participants. Approximately 59% of respondents reported using at least one AI tool or application on a daily basis. Furthermore, the majority of respondents agreed that digital medical services, mentioned in a previous question, could be beneficial in reducing unnecessary interactions between patients and healthcare providers.

Conclusion: The COVID-19 pandemic has demonstrated the transformative potential of AI in pandemic response. AI has played a crucial role in various aspects of combating COVID-19, including patient diagnosis, treatment development, and vaccine creation. However, challenges and limitations exist in terms of data accessibility, bias, and privacy when utilizing AI. These issues need to be addressed to ensure the ethical and responsible use of AI in the fight against COVID-19 and future pandemics.

## Introduction

Almost every aspect of life has been impacted by the COVID-19 pandemic. The pandemic has significantly altered the world we live in, affecting everything from healthcare systems and economies to social interactions and everyday routines. The use of artificial intelligence (AI) is one of the main ways that technology has contributed to the pandemic. AI has the power to completely change how we identify, monitor, and react to pandemics, such as COVID-19 [1].

Using algorithms and machine learning to analyze data and make predictions or judgments is referred to as AI. To find patterns and forecast the spread of the virus in the context of the COVID-19 pandemic, AI can be used to evaluate vast amounts of data from numerous sources, including electronic health records, social media, and news reports. AI can be used to create and improve medications and vaccines and to monitor the success of public health initiatives [2]. AI has emerged as a powerful tool in the field of pharmaceutical research, offering innovative avenues for the creation and optimization of medications. This scientific exploration delves into the diverse applications of AI in drug development, highlighting its potential to revolutionize the drug discovery process and enhance therapeutic efficacy. AI techniques, such as machine learning, deep learning, and data mining, enable the analysis of vast amounts of biomedical data, including molecular structures, genomic profiles, and clinical trial outcomes. By harnessing AI algorithms, researchers can identify novel drug targets, predict compound properties, optimize chemical structures, and generate virtual drug libraries for in silico screening. This accelerates the identification of potential drug candidates with improved efficacy, reduced side effects, and enhanced pharmacokinetic profiles.

This technology can be used to monitor the success of public health initiatives. For instance, AI can be used to monitor compliance with social distancing policies and mask use by analyzing data from social media. To detect possible outbreaks and assess the success of contact-tracing efforts, AI can also be used to analyze data from contact-tracing apps. The fight against COVID-19 has greatly benefited from research, and the number of COVID-19-related AI models appearing in papers is rising quickly. AI models that have been properly trained can help physicians diagnose patients more quickly and accurately while also requiring less manual work [3-4]. AI models could characterize COVID-19 epidemiology, identify patients at greater risk for the disease, and model disease transmission using training data [5-6]. Utilizing AI, researchers may be able to repurpose existing medications, identify possible vaccine targets based on SARS-CoV-2 mutation models, and identify compounds that could be used as vaccine adjuvants [7]. A lot more people can get advice from AI-powered chatbots than from manned contact centers, which relieves pressure on medical hotlines and has been successfully used in clinical scenarios [8]. AI could control the pandemic by employing thermal imaging to search public areas for individuals who may be infected and by imposing social segregation and lockdown procedures [7].

In many areas of the COVID-19 pandemic, such as diagnosis, public health, clinical decision-making, social control, therapeutics, vaccine development, surveillance, combination with big data, operation of other key clinical services, and management of COVID-19 patients, AI has been extensively used [7-8]. The most crucial steps to stopping the spread of the COVID-19 pandemic are quick diagnosis, accurate prediction, enhanced monitoring, and efficient treatments. This will relieve the substantial strain on the scarce medical resources that have been brought on by the pandemic. Hence, by means of this cross-sectional study, we aimed to analyze knowledge, awareness, and perceptions about the COVID-19 pandemic and its correlation with AI among the general Saudi Arabian population. The other objectives that were assessed included the perceptions of general health and well-being and the individual acceptance of AI by the respondents.

## Materials and methods

Study design

The study's methodology, sampling, data-gathering techniques, and analysis were all based on a cross-sectional survey design. The strategy involved gathering information and drawing conclusions over a specific period [9]. In this instance, the study was performed in Saudi Arabia with a focus on the population of Saudis residing in the north, south, east, west, and central areas. A percentage of 95.7% of Saudis, the group of people the researchers focused on, had access to the Internet [10].

Study sample

Saudi nationals made up the study's target group [10]. People who were over 18 and ready to participate in the study met the inclusion criteria. To get a comprehensive view of the effectiveness of the efforts to stop the spread of COVID-19, foreigners living in the country were excluded from the research. By contrast, the researchers used a purposive method in which only respondents who met the criteria for inclusion took part in the study. Using the Raosoft online survey size calculator (Raosoft, USA) [11] and considering the average population of Saudi Arabia [10], we estimated that a sample size of around 270 (determined by a 90% confidence interval and a 5% margin of error) would be sufficient for the purposes of this study.

Questionnaire protocol

The main study tool that we used was an online questionnaire. The instrument was modified to fit the goals of this research after being modified for use in a prior study. The previous research concentrated on knowledge, attitudes, and practices (KAP) in relation to the general perception of the COVID-19 pandemic, which was at its height at the time among people in our area [12]. The survey had four parts, the first of which contained four items with demographic information. The focus of the second part was on knowledge, the third on attitude, and the fourth on practices. As the researcher compared the primary data findings with those of earlier scholars on a related subject, literature reviews were the main source of secondary data. To accommodate the interviewees' preferred languages, the questionnaire was made available in both Arabic and English.

The survey was conducted solely through online channels to reach the target population. An online survey platform was utilized to design and host the survey questionnaire. The survey was then disseminated using various online channels and strategies to maximize participation. Email invitations were sent to potential participants, either directly or through targeted mailing lists, explaining the purpose of the survey and providing a link to access the online questionnaire. The email invitations emphasized the importance of the participants' responses and encouraged them to complete the survey. In addition, social media platforms played a significant role in distributing the survey. Announcements and reminders about the survey were shared on social media platforms, such as Facebook, Twitter, LinkedIn, or relevant online communities and forums. These posts included a brief description of the survey, its significance, and a direct link to the online questionnaire.

Statistical protocol

A descriptive statistic of the frequency distribution and percentages was calculated for the categorical variables. The relationship between the categorical variables of questionnaire items and demographic variables was assessed using the chi-square test. A p-value of 0.05 was considered statistically significant. All data were analyzed using statistical software (SPSS, released 2017; IBM Corp., Armonk, New York, United States).

Ethical protocol

The study underwent due compliance as per the ethical requirements of the Institutional Review Board (IRB) at Riyadh Elm University. As a result, our investigation was approved with the IRB approval number SRP/2021/70/476/449.

## Results

Out of the 270 individuals that seemed to be sufficient for our investigation, we received 153 responses, indicating a response rate close to 57%. We aimed to analyze the distribution of the age of our respondents in four different stratifications: 20-29, 30-39, 40-49, and 50-59 year olds. The observations received showed that 34.6% of the participants, i.e., the majority of the respondents, were in the 30-39-year-old group. Females comprised nearly 55% of the sample size. Table 1 demonstrates the demographic characteristics of these respondents.

**Table 1:** Characteristics of the study participants

Variables		N	%
Nationality	Saudi	51	32.5%
	Non-Saudi	102	67.5%
Gender	Male	69	45.1%
	Female	84	54.9%
Age group	20-29	20	13.1%
	30-39	53	34.6%
	40-49	48	31.4%
	50-59	32	20.9%
Region	North	10	6.5%
	South	49	32.0%
	East	21	13.7%
	West	21	13.7%
	Central	52	34.0%

Table 2 shows the mean and SD values for the respondents when they answered the questionnaire in the "Awareness and Concern" category. It also displays that the majority of the individuals (67.3%) believed they would contract the coronavirus at some point, and 15% answered they most definitely would.

**Table 2:** COVID-19: Awareness and concern SD: Standard deviation

Questions		Mean		SD	Minimum	Maximum	Count	% (percentage)
On a scale of 1 to 10, how serious of a public health threat do you think the COVID-19 is or might become? (1 being 2 threat at all, 10 being a very serious public health threat)		8.19		2.22	0	10	-	-
How worried are you about getting the COVID-19?		7.43		2.75	0	10	-	-
How likely it is that someone you know may get sick from the COVID-19 this year because they do not follow the guidelines?		5.67		3.12	0	10	-	-
Do you think that you will get sick from the COVID-19?	I definitely will	-		-	-	-	23	15.00%
	I probably will	-	-		-	-	103	67.30%
	Neutral	-	-		-	-	27	17.60%

The present results from a survey on the awareness of asymptomatic COVID-19 carriers and their perception of the likelihood of displaying no symptoms or mild symptoms and death due to COVID-19. Of the respondents, 90.8% were aware that COVID-19 carriers can be asymptomatic, while 5.9% were not aware and 3.3% were unsure. The mean score for the likelihood of displaying no symptoms or mild symptoms on a scale of 1 to 10 was 7.00, with a standard deviation (SD) of 2.27, indicating a relatively high level of belief that infected individuals can be asymptomatic or exhibit mild symptoms. On the other hand, the mean score for the likelihood of dying from COVID-19 was 4.50, with an SD of 2.39, indicating a lower level of belief in the severity of the disease. The minimum score for both questions was 0, indicating no likelihood, while the maximum score was 10, indicating the highest level of likelihood. Overall, the results suggest that a majority of the respondents are aware of asymptomatic carriers, and most believe that infected individuals can display no symptoms or mild symptoms. However, there is a relatively low level of belief in the severity of the disease, as the mean score for the likelihood of death due to COVID-19 is relatively low, as shown in Table 3.

**Table 3:** COVID-19: Knowledge SD: Standard deviation

Questions		Count	% (percentage)	Mean	SD	Minimum	Maximum
Are you aware that COVID-19 carriers can be asymptomatic? For example, absence of running nose, cough, fever or appearing to be fine.	Yes	139	90.80%	-	-	-	-
	No	9	5.90%	-	-	-	-
	Unsure	5	3.30%	-	-	-	-
On a scale of 1 to 10, how likely do you think a person who is infected by COVID-19 will display no symptoms or mild symptoms? For example, mild cough, itchy throat, and mild fever.		-	-	7	2.27	0	10
On a scale of 1 to 10, how likely do you think a person who gets COVID-19 will die as a result of COVID-19?		-	-	4.5	2.39	0	10

The majority of the respondents (96.1%) reported that they wear a mask whenever they are outside of their house. Similarly, 93.5% of the participants reported that they keep a minimum distance of 1 m from others in public, and 94.8% reported that they wash their hands frequently. The majority of the respondents (79.7%) also reported that they stay at home as much as possible. In addition, 92.2% of the respondents reported that they avoid touching their face, nose, and mouth. The proportion of respondents who reported that they do not follow the recommended prevention measures was relatively low, with the highest percentage being 8.5% for staying at home as much as possible. The proportion of respondents who were unsure about following the recommended prevention measures was also relatively low, with the highest percentage being 11.8% for staying at home as much as possible. Overall, the results suggest that a majority of the respondents are following the recommended COVID-19 prevention measures, with a low proportion reporting that they are not following the measures or are unsure about them. Table 4 presents the responses of the survey participants to questions about COVID-19 prevention measures.

**Table 4:** Prevention methods against COVID-19

Questions		n	%
Wear a mask (as long as you are outside of the house)	Yes	147	96.1%
	No	4	2.6%
	Unsure	2	1.3%
Keep a minimum distance of 1 m from others in the public	Yes	143	93.5%
	No	6	3.9%
	Unsure	4	2.6%
Wash your hands frequently	Yes	145	94.8%
	No	3	2.0%
	Unsure	5	3.3%
Stay at home as much as possible	Yes	122	79.7%
	No	13	8.5%
	Unsure	18	11.8%
Avoid touching your face, nose, and mouth	Yes	141	92.2%
	No	5	3.3%
	Unsure	7	4.6%

The majority of the respondents (73.9%) reported that they are either very prepared or somewhat prepared for a further outbreak. Only a small percentage of the respondents (6.6%) reported that they were not very well prepared or not prepared at all. In terms of the impact of the pandemic on their daily routine, 65.4% of the respondents reported that it has changed their routine a lot, while 28.1% reported a moderate change. The majority of the respondents (73.9%) reported that the pandemic has caused them to lose sleep, with 20.9% of respondents reporting rather more than usual or much more than usual sleep loss. In addition, the majority of the respondents (66.7%) reported feeling more stressed than usual due to the pandemic. Only a small percentage of the respondents (7.8%) reported feeling less stressed than usual. Overall, the results suggest that the majority of the respondents feel prepared for a further outbreak, but the pandemic has had a significant impact on their daily routine, sleep patterns, and stress levels. Table 5 presents the results of a survey on the perceived level of preparedness for a further widespread outbreak in the local community and the impact of the COVID-19 pandemic on the daily routine, sleep patterns, and stress levels.

**Table 5:** Perception of the COVID-19 pandemic on general health and well-being

Questions		n	%
How prepared do you think you are if there were to be a further widespread outbreak in the local community?	Very prepared	50	32.7%
	Somewhat prepared	63	41.2%
	Neutral	30	19.6%
	Not very well prepared	9	5.9%
	Not prepared at all	1	0.7%
How much has the COVID-19 pandemic changed your daily routine?	A lot	100	65.4%
	Moderate	43	28.1%
	A little	9	5.9%
	Not at all	1	0.7%
How much has the COVID-19 pandemic caused you to lose sleep?	Less than usual	44	28.8%
	No more than usual	69	45.1%
	Rather more than usual	32	20.9%
	Much more than usual	8	5.2%
How much has the COVID-19 pandemic caused you to feel under stress?	Less than usual	12	7.8%
	More than usual	102	66.7%
	Neutral	39	25.5%

The results of the survey on the opinions and likelihood of using digital medical services and automated software/AI systems for medical diagnosis and advice during the COVID-19 pandemic are shown in Table 6.

**Table 6:** Items of AI

Questions		n	%
Do you agree that the digital medical services mentioned in the previous question may be helpful to reduce non-essential contact between patients and doctors/healthcare providers?	Yes	123	81.5%
	No	13	8.6%
	Unsure	15	9.9%
If the COVID-19 pandemic continues, how likely will you use these digital medical services (video consultation with doctors, WhatsApp chats/SMS text))	Very likely	75	49.7%
	Somewhat likely	48	31.8%
	Neutral	19	12.6%
	Continued	1	0.7%
	Not at all likely	8	5.3%
If the COVID-19 pandemic continues, will you feel comfortable using automated software/AI systems to interpret your medical tests/scans and provide advice automatically?	Very comfortable	55	36.4%
	Somewhat comfortable	41	27.2%
	Neutral	35	23.2%
	Somewhat uncomfortable	9	6.0%
	Not at all comfortable	11	7.3%

The majority of the respondents (81.5%) agreed that digital medical services could be helpful in reducing non-essential contact between patients and healthcare providers. When asked about the likelihood of using digital medical services if the pandemic continues, almost 81.5% of the respondents reported that they are either very likely or somewhat likely to use these services. However, 5.3% of the respondents reported that they were not at all likely to use these services. When asked about the use of automated software or AI systems to interpret medical tests and scans, a significant percentage of the respondents (63.6%) reported feeling comfortable with this technology, either very comfortable or somewhat comfortable. However, a small percentage of the respondents (13.3%) reported feeling uncomfortable or not at all comfortable with this technology. Overall, the results suggest that a large percentage of the respondents believe that digital medical services could be useful in reducing non-essential contact, and a majority of the respondents reported feeling comfortable using automated software or AI systems for medical diagnosis and advice during the COVID-19 pandemic.

Of the respondents, 58.3% reported using AI applications to monitor their symptoms, with 41.7% reporting that they did not. Of those who did use the applications, 41.7% logged in every day, 19.9% logged in less often, and 38.4% logged in only when asked. The majority of the respondents, 76.2%, reported feeling safer using these applications. Regarding specific applications, 49.7% of the respondents were aware that the Tawakkalna app (Saudi Data and Artificial Intelligence Authority (SDAIA), Saudi Arabia) can monitor a mobile phone even if it is turned off. Moreover, 92.1% of the respondents believed that these applications would help prepare us in the future to reduce the spread of infectious diseases other than COVID-19. Finally, 73.5% of the respondents agreed that Tawakkalna is the main reason for the decreasing number of cases caused by gatherings. The table also presents the results of the survey regarding the use of government-managed health applications, their impact on people's behavior, and the spread of COVID-19. The majority of the respondents (89.4%) agreed that these applications have helped make people more careful and committed. Moreover, most participants (82.8%) reported that they had installed all the applications managed by the Department of Health. A large number of respondents (71.5%) thought that the applications were not only related to infected people. Furthermore, a majority of the participants (80.8%) knew that Tabaud (SDAIA, Saudi Arabia) notifies users if they have been in contact with people who have tested positive for COVID-19. The majority of the respondents (73.5%) believed that SEHA’s (Abu Dhabi Health Services Company, UAE) online medical consultation services reduced the spread of COVID-19. However, some respondents expressed concerns about privacy and security threats (24.5%), while others were uncertain (40.4%). Finally, the participants had mixed views on whether these applications had reduced the number of infected individuals, with 53.6% agreeing, 6.6% disagreeing, and 39.7% unsure. Overall, the survey suggests that these government-managed health applications have had a positive impact on people's behavior and have been helpful in reducing the spread of COVID-19, although there are still concerns about privacy and security. Table 7 presents data on the use of AI applications to monitor symptoms and attitudes toward them, as well as awareness of specific applications.

**Table 7:** Use of applications

Questions		n	%
Are you using any of the AI applications to monitor your symptoms?	Yes	88	58.3%
	No	63	41.7%
How often do you log into the application?	Everyday	63	41.7%
	Not so often	30	19.9%
	Only when asked	58	38.4%
Do you feel safer using applications as opposed to not?	Yes	115	76.2%
	No	36	23.8%
Do you know Tawakkalna app can monitor the mobile phone even if it was turned off?	Yes	75	49.7%
	No	76	50.3%
Do you think these applications will help to prepare us in the future to reduce the spread of infectious diseases other than COVID-19?	Yes	139	92.1%
	No	12	7.9%
Do you know someone that does not know about Tawakkalna, SEHA, and Tabaud applications?	Yes	40	26.5%
	No	111	73.5%
Do you know the applications from the Ministry of Health that helped you know about COVID-19 more?	Yes	122	80.8%
	No	29	19.2%
Do you think Tawakkalna is the main reason for decreasing the number of cases caused by gathering?	Yes	111	73.5%
	No	40	26.5%
Do you think these applications has helped make people to be more careful and committed?	Yes	135	89.4%
	No	16	10.6%
Did you install all the applications that are managed by the Ministry of Health?	Yes	125	82.8%
	No	26	17.2%
Do you think the application(s) are related to infected people only?	Yes	21	13.9%
	No	108	71.5%
	May be	22	14.6%
Did you know that Tabaud notifies you if you have been in contact with people who have tested positive, as it enables each user to receive direct and proactive notifications if any registered infected person has been detected around them?	Yes	122	80.8%
	No	29	19.2%
Do you think that “SEHA” online medical consultation services was helpful?	Yes	93	61.6%
	No	58	38.4%
Do you think “SEHA” that provides online medical consultation services reduced the spread of COVID-19?	Yes	111	73.5%
	No	40	26.5%
Do you think your privacy and security will be threatened if you install these applications?	Yes	37	24.5%
	No	53	35.1%
	May be	61	40.4%
Do you think that after launching these applications, the number of infected individuals was reduced?	Yes	81	53.6%
	No	10	6.6%
	May be	60	39.7%
Are you aware of all the services that are provided by the Ministry of Health?	Yes	87	57.6%
	No	19	12.6%
	Unsure	45	29.8%
Do you rely on these applications if you needed any information or medical service?	Yes	101	66.9%
	No	50	33.1%
Do you think these applications promote a healthy lifestyle through various features for the future?	Very likely	80	53.0%
	Somewhat unlikely	32	21.2%
	Neutral	36	23.8%
	Not at all likely	3	2.0%

The "Yes" column indicates the percentage of respondents who answered affirmatively to the question, while the "No" column represents the percentage who answered negatively. The table also includes the p-values, which assess the statistical significance of the differences observed. The data shows that the majority of non-Saudi respondents rely on these applications for information or medical services (69.3%), compared to only 30.7% of Saudis. However, this difference was not statistically significant (p = 0.512). Gender also appears to be associated with reliance on these applications, with 50.5% of male respondents indicating they rely on them, compared to only 49.5% of females. However, this difference was not statistically significant either (p = 0.092). Age groups were also examined, and the results show that those in the 30-39-year-old age group (37.6%) were most likely to rely on these applications, followed by the 40-49-year-old age group (31.7%). The differences between age groups, however, were not statistically significant (p = 0.712). Regarding the region, the respondents in the south (39.6%) were most likely to rely on these applications for information or medical services, while those in the central region (46.0%) were least likely to do so. The p-value for the region was marginally significant (p = 0.077), indicating that there may be some regional differences in reliance on these applications. Table 8 presents the results of a survey regarding the reliance on applications for information or medical services, broken down by nationality, gender, age group, and region.

**Table 8:** Assessment on individual acceptance toward AI, pre- and post-COVID-19

Do you rely on these applications if you needed any information or medical service?				
		Yes	No	p
		%	%	
Nationality	Saudi	30.7%	36.0%	0.512
	Non-Saudi	69.3%	64.0%	
Gender	Male	50.5%	36.0%	0.092
	Female	49.5%	64.0%	
Age group	20-29	11.9%	12.0%	0.712
	30-39	37.6%	30.0%	
	40-49	31.7%	32.0%	
	50-59	18.8%	26.0%	
Region	North	5.9%	4.0%	0.077
	South	39.6%	18.0%	
	East	12.9%	16.0%	
	West	12.9%	16.0%	
	Central	28.7%	46.0%	

**Figure 1:**
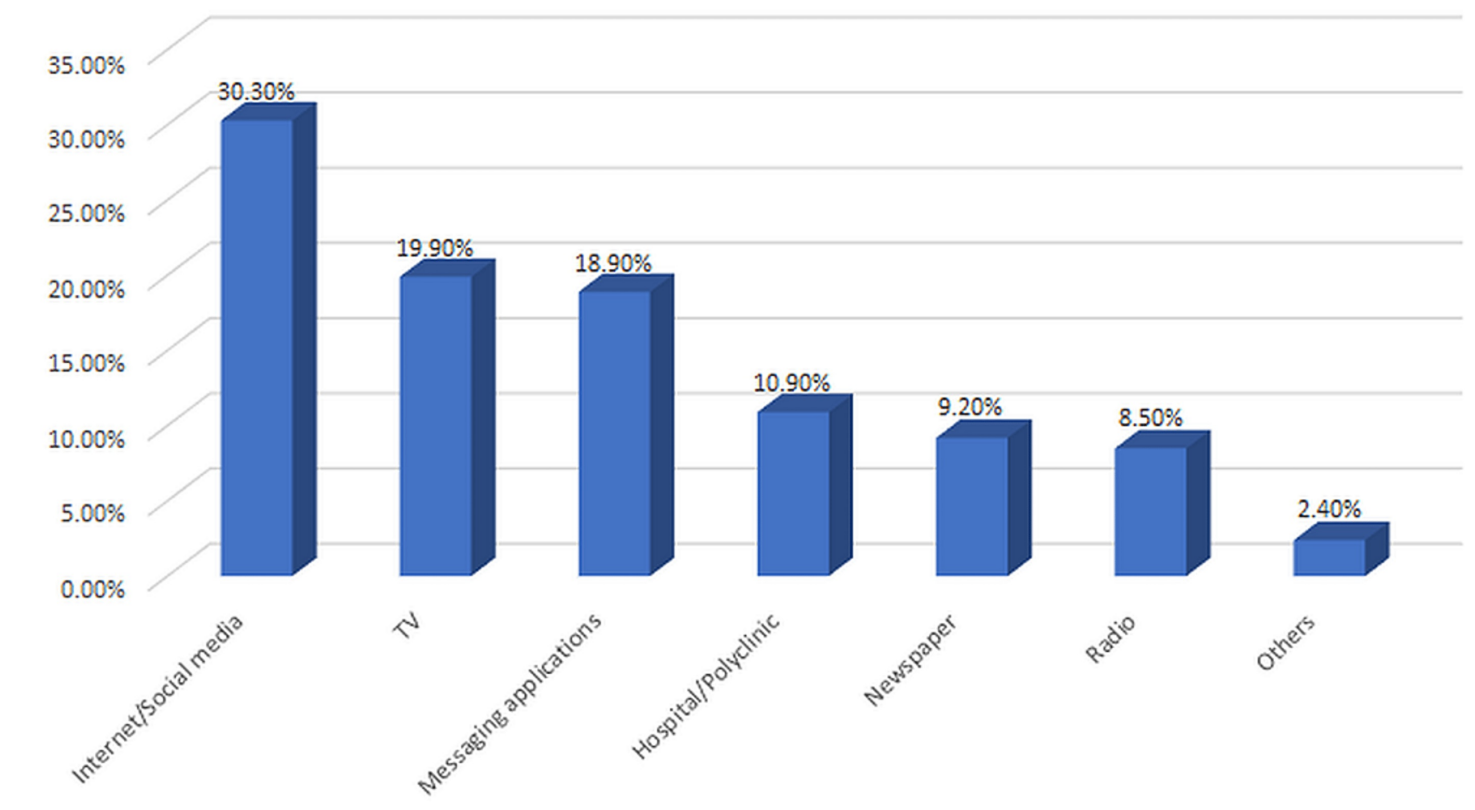
Source of information for COVID-19 that the respondents have used

Figure 1 shows the sources of information for COVID-19 that the respondents have used, with the majority relying on online sources, such as social media, news websites, and search engines.

**Figure 2:**
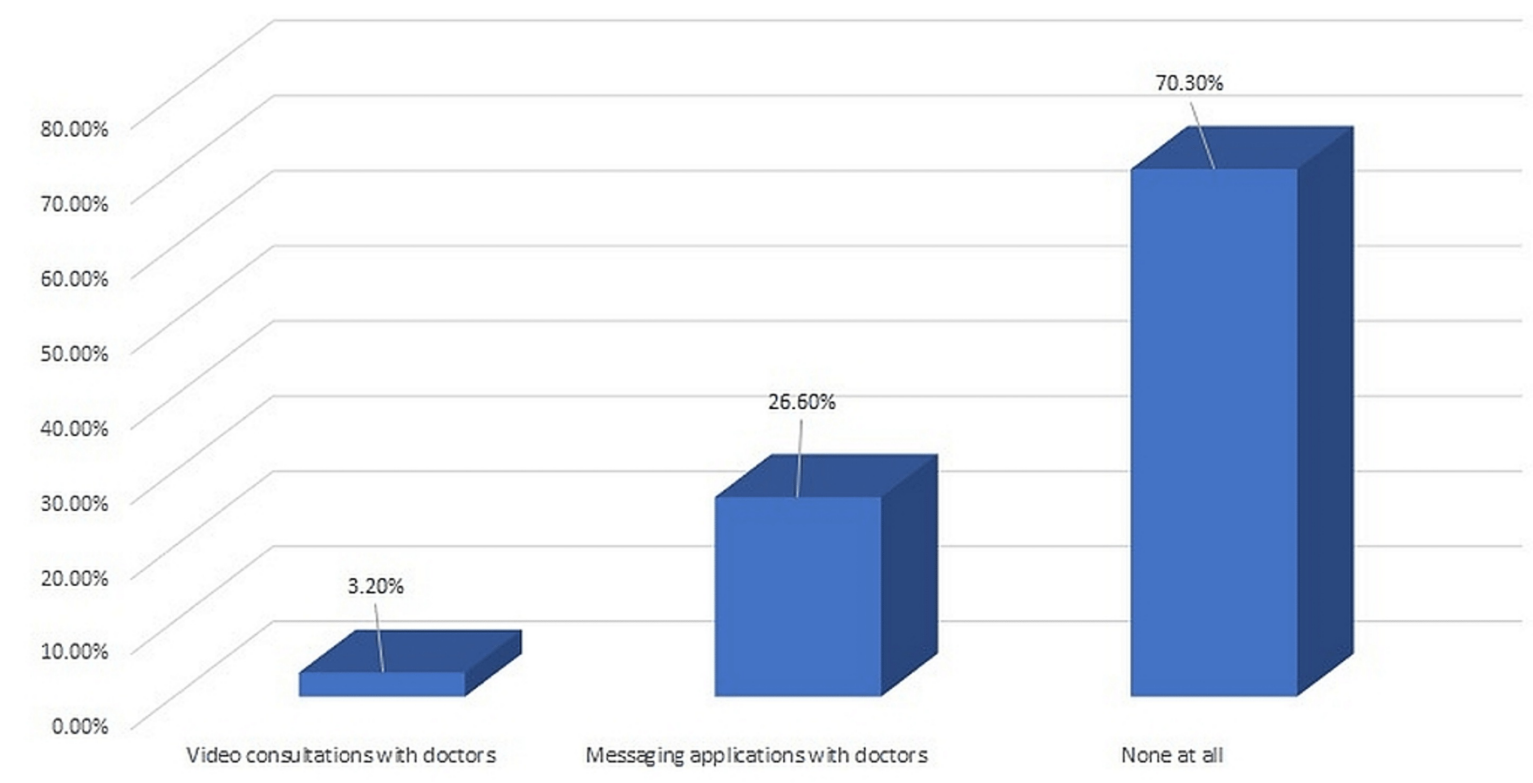
Usage pattern of AI methodologies

Only a small percentage of the respondents have relied on healthcare providers for COVID-19 information. Figure 2 shows the usage pattern of AI methodologies.

**Figure 3:**
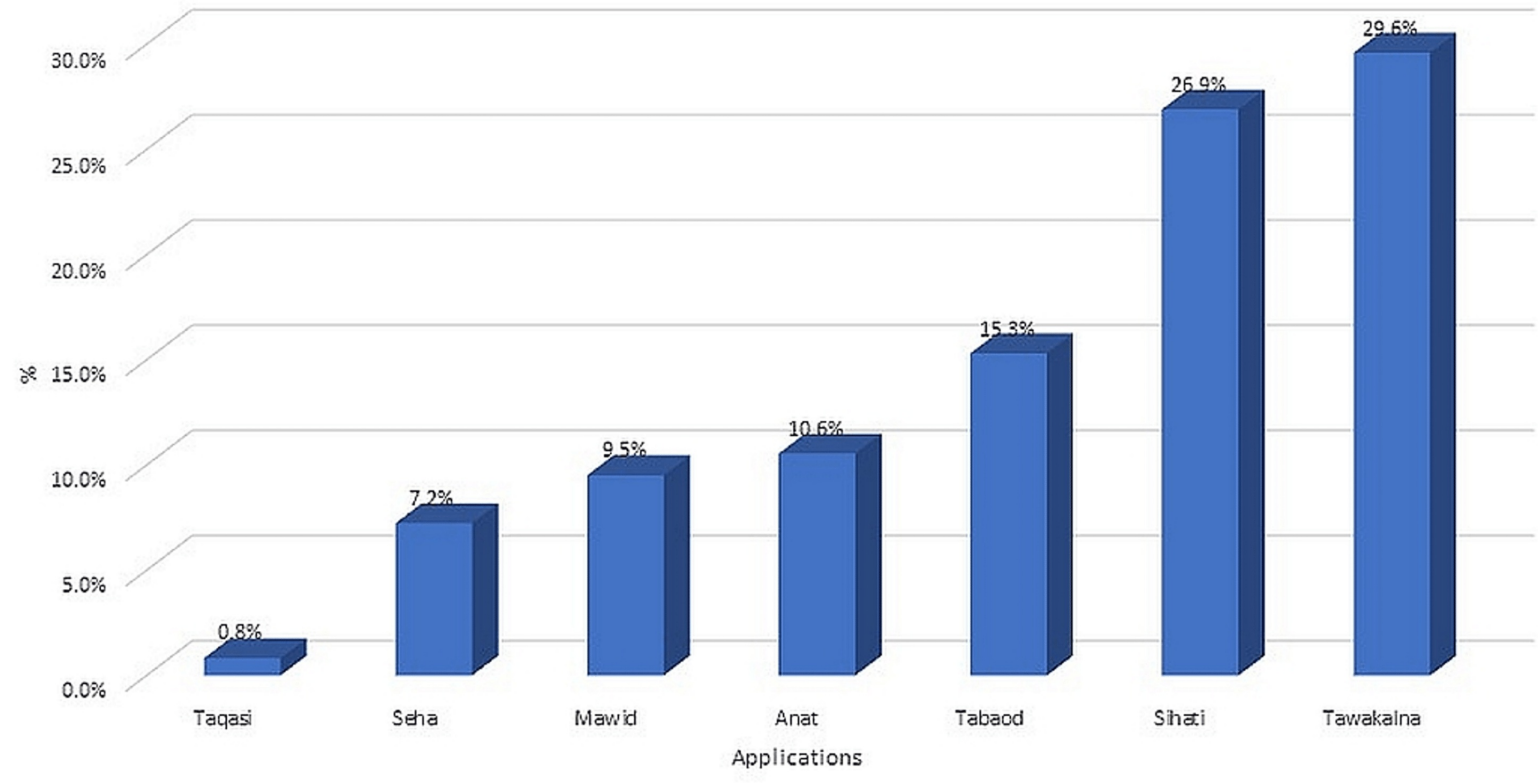
AI applications that the respondents are using

Figure 3 shows the various AI applications that the respondents are using.

The most commonly used application is the "Tawakkalna" app, followed by the "SEHA" app. Other AI applications mentioned by the respondents include "Tabaud" and "Mawid" apps. The most commonly used feature in these applications is the ability to check for COVID-19 symptoms, followed by the ability to receive notifications of exposure to infected individuals. Other features mentioned by the respondents include online medical consultations and the ability to book vaccination appointments.

## Discussion

AI has the potential to revolutionize the pandemic response by enabling faster and more accurate diagnosis, more effective treatments and vaccines, and more targeted public health interventions [13]. However, there are also challenges and limitations to the use of AI in pandemic response. One challenge is the availability and quality of data. AI algorithms rely on large amounts of high-quality data to make accurate predictions and decisions. In the case of COVID-19, there have been challenges with data collection and sharing, particularly in the early stages of the pandemic. Another challenge is the potential for bias in AI algorithms. AI algorithms are only as good as the data they are trained on. If the data are biased, for example, if it underrepresents certain populations or regions, the AI algorithm may make inaccurate predictions or decisions. Finally, there are ethical and privacy concerns with the use of AI in pandemic responses. For example, the use of AI to track compliance with public health measures may raise concerns about privacy and surveillance [14].

Zhang et al. [13] created a protein three-dimensional (3D) model of 3-chymotrypsin like protease (3CLPro), identified protein-ligand interacting pairs using deep learning, and then gave lists of potential compounds and tripeptides for 3CLpro. Batra et al. [15] used high-fidelity ensemble docking and machine learning to extract 75 FDA-approved and 100 additional ligands as possible COVID-19 therapeutic agents from drug datasets. Deep-learning models were used by Joshi et al. [16] to screen natural compounds. They discovered that two of the compounds made a very stable complex that could be used to develop new antiviral treatments for SARS-CoV-2. To screen 1.3 billion compounds from the ZINC15 library and find the top 1,000 potential ligands for the SARS-CoV-2 protein, Ton et al. [17] created Deep Docking (DD). Globally, COVID-19 has spread and had a sizable effect. In March 2020, the World Health Organization (WHO) classified it as a pandemic. Alsayed et al. [18] predicted the course of the pandemic or calculated the unreported number of infections using the susceptible-exposed-infectious-recovered (SEIR) model in conjunction with machine learning. It is crucial to concentrate on developing prediction models as the COVID-19 pandemic develops to aid lawmakers and health managers in allocating healthcare resources and preventing or limiting outbreaks [19].

Multilayer perceptron (MLP) artificial neural networks were put to the test by Mollalo et al. [20] in their attempt to simulate the cumulative prevalence of COVID-19 at the county level across the United States. In order to predict confirmed cases, deaths, and recoveries in 10 major countries affected by COVID-19, Shahid et al. [21] proposed prediction models that included support vector regression (SVR), autoregressive integrated moving average (ARIMA), long short-term memory (LSTM), and bi-directional long short-term memory (Bi-LSTM). An enhanced susceptible-infected (ISI) model was put forth by Zheng et al. [22] to analyze transmission patterns and development trends and determine the range of infection rates. In order to predict the cumulative confirmed cases of COVID-19 in the 10 Brazilian states with the highest daily incidence, Ribeiro et al. [23] used a variety of machine learning algorithms, ranking them according to their level of accuracy. The outcomes of these studies may help to control and prevent COVID-19, which could have wide-ranging positive effects. AI and its knowledge, awareness, and perception among the Saudi Arabian population shed light on the understanding and acceptance of this transformative technology in the context of combating COVID-19. The findings provide valuable insights into the attitudes and behaviors of individuals toward AI and its applications in healthcare.

One notable finding from the studies is the age distribution of the participants. The majority of the respondents fell within the age range of 30 to 39 years. This suggests that younger individuals are more likely to engage with and have a higher level of familiarity with AI. This could be attributed to the digital native nature of younger generations, who have grown up in a technologically advanced era. The predominance of female participants in the surveyed population is another interesting aspect. This could be indicative of a greater interest and engagement of women in healthcare-related topics and emerging technologies. It also highlights the importance of gender inclusivity in the discussions and implementation of AI solutions.

The fact that almost 59% of the respondents reported using at least one AI tool or application daily reflects the increasing integration of AI into everyday life [21]. This indicates that AI technologies are becoming more accessible and widely adopted by the Saudi Arabian population. The use of AI tools and applications could range from voice assistants and smart home devices to health-related apps and wearable technologies. The acceptance and utilization of AI in daily routines reflect a positive perception and confidence in the potential benefits offered by AI. These study results highlight the significance of AI in revolutionizing pandemic responses, particularly in the case of COVID-19. AI has demonstrated its potential in various aspects, such as early detection, diagnosis, treatment development, and vaccine creation. The positive perception and acceptance of AI among the Saudi Arabian population suggest a readiness to embrace and leverage technological advancements in healthcare.

Some limitations do exist with respect to our investigation. Because the researchers used an online questionnaire, not every Saudi had the same opportunity to take part as 5% of the population does not have access to the Internet [10]. As a consequence, the findings might not apply to the entire Saudi Arabian population. In addition, this research was a cross-sectional study carried out at a particular time, and viewpoints may change.

## Conclusions

The COVID-19 pandemic has demonstrated the potential of AI to transform the pandemic response. From diagnosing patients to developing treatments and vaccines, AI has played a critical role in the fight against COVID-19. However, there are also challenges and limitations to the use of AI, particularly around data availability, bias, and privacy. As the world continues to grapple with the pandemic and prepare for future pandemics, it will be important to address these challenges and harness the full potential of AI to improve public health outcomes.
